# Immune System and Epidemics: The Role of African Indigenous Bioactive Substances

**DOI:** 10.3390/nu15020273

**Published:** 2023-01-05

**Authors:** Chiara Frazzoli, Gerardo Grasso, Danladi Chiroma Husaini, Doris Nnenna Ajibo, Fortune Chiemelie Orish, Orish E. Orisakwe

**Affiliations:** 1 Department for Cardiovascular, Endocrine-Metabolic Diseases, and Aging, Istituto Superiore di Sanità, 00162 Rome, Italy; 2 Istituto per lo Studio dei Materiali Nanostrutturati Sede Sapienza, Consiglio Nazionale delle Ricerche, P.le Aldo Moro 5, 00185 Rome, Italy; 3 Department of Pharmacology & Toxicology, Faculty of Health Sciences, University of Belize, Belmopan P.O. Box 430, Belize; 4 Department of Experimental Pharmacology & Toxicology, Faculty of Pharmacy, University of Port-Harcourt, Port-Harcourt 5323, Nigeria; 5 Provictoire Research Institute, Port-Harcourt 234, Nigeria; 6 African Centre of Excellence for Public Health and Toxicological Research (ACE-PUTOR), University of Port Harcourt, PMB, Port Harcourt 5323, Nigeria

**Keywords:** pandemics, epidemics, immune system, immune boosters, bioactive compounds, fermented foods, herbal remedies, medicinal plants, Africa

## Abstract

With over 6 million coronavirus pandemic deaths, the African continent reported the lowest death rate despite having a high disease burden. The African community’s resilience to the pandemic has been attributed to climate and weather conditions, herd immunity, repeated exposure to infectious organisms that help stimulate the immune system, and a disproportionately large youth population. In addition, functional foods, herbal remedies, and dietary supplements contain micronutrients and bioactive compounds that can help boost the immune system. This review identified significant traditional fermented foods and herbal remedies available within the African continent with the potential to boost the immune system in epidemics and pandemics. Methodology: Databases, such as PubMed, the Web of Science, and Scopus, were searched using relevant search terms to identify traditional African fermented foods and medicinal plants with immune-boosting or antiviral capabilities. Cereal-based fermented foods, meat-, and fish-based fermented foods, and dairy-based fermented foods containing antioxidants, immunomodulatory effects, probiotics, vitamins, and peptides were identified and discussed. In addition, nine herbal remedies and spices belonging to eight plant families have antioxidant, immunomodulatory, anti-inflammatory, neuroprotective, hepatoprotective, cardioprotective, and antiviral properties. Peptides, flavonoids, alkaloids, sterols, ascorbic acid, minerals, vitamins, and saponins are some of the bioactive compounds in the remedies. Bioactive compounds in food and plants significantly support the immune system and help increase resistance against infectious diseases. The variety of food and medicinal plants found on the African continent could play an essential role in providing community resilience against infectious diseases during epidemics and pandemics. The African continent should investigate nutritional, herbal, and environmental factors that support healthy living and longevity.

## 1. Introduction

The coronavirus pandemic recorded a high incidence of infections, hospitalizations, and deaths globally. As of May 2022, over 6 million people have died due to the coronavirus infection worldwide, with the African continent accounting for less than a million deaths, making the continent have the lowest death count due to the COVID-19 pandemic [1]. Approximately 25% of the global disease burden had been reported in Africa before the coronavirus. An estimated 2.4 trillion USD is spent annually in the region due to a high prevalence of communicable and non-communicable diseases, injuries, and trauma [2]. In addition, lack of resources, absence or inadequate modern health facilities, lack of access to health facilities, corruption and bad governance, harmful cultural practices, and environmental factors added to the challenges of sustainable health development in the continent [3,4]. Despite these overwhelming disease challenges in Africa, a lower prevalence of COVID-19 deaths was reported. Climate and weather conditions, herd immunity, repeated exposure to infectious organisms that help stimulate the immune system, and a disproportionately large youth population have been put forward as possible reasons for the lower death rates of COVID-19 [5]. Furthermore, due to the fear of acquiring COVID-19, African healthcare workers occasionally forsook their posts, leaving some to die from other diseases, such as stroke, diabetes, cardiovascular diseases, and malaria, as they had no easy access to therapy. Thus, a proportion of the excess deaths during the pandemic were not directly connected to COVID-19 but partly to medical negligence and mismanagement [5].

In addition, the disease prevention of various types of disorders, such as cardiovascular diseases, obesity, and infectious diseases, has been appropriately linked to correct eating habits, adequate diet, and exercise [6]. The efficient performance of the immune system is fundamental to the prevention and sustenance of the body; hence, good nutrition derived from food components is fundamental in boosting the immune system. Functional foods, herbal remedies, and dietary supplements contain micronutrients and bioactive compounds that can help boost the immune system. Micronutrients and bioactive natural substances can boost communities’ resilience to contagious epidemics in Africa and around the world. Micronutrients in foods, plants, and dietary supplements are widely acceptable, available, affordable, and accessible by the general population in Africa. Furthermore, micronutrients in food and plants contain antioxidant, anti-inflammatory, cardioprotective, and immunomodulatory activities that might have contributed to the lower rates of deaths reported on the African continent due to COVID-19. Therefore, foods, food ingredients, and plants are significant in preventing and treating infections during pandemics and their health outcomes. Undoubtedly, in addition to good nutritional diets, hygiene practices and vaccinations are still the most effective recommended public health measures against all viral infections [7]. We conducted this review to identify significant African traditional fermented foods and herbal remedies that are acceptable, available, affordable, and accessible with the potential for use in epidemics and pandemics. We presented their traditional uses, active ingredients, pharmacologic activity, and potential for providing community resilience during epidemics and pandemics. The review is intended to motivate the exploitation of health benefits from traditional African fermented foods and bioactive compounds found in medicinal plants for future epidemics and pandemics, taking into consideration the general population, subgroups, and regional variations within the African subcontinent. 

## 2. Literature Search Methodology

We searched PubMed, Web of Science, Scopus, JSTOR, Google Scholar, and ScienceDirect databases to identify peer-reviewed research, books, review articles, and articles that examined traditional fermented foods and herbal remedies used in epidemics and pandemics within the African continent. Articles and publications that evaluated “fermented foods as immune boosters,” “fermented cereal,” fermented meat,” “fermented fish,” “fermented dairy products,” “immune boosters,” “pandemics,” “epidemics,” and “treatment of viral infections,” “micronutrients,” “bioactive compounds,” “prevention of disease,” “community health resilience,” “herbal remedies,” “medicinal plants,” traditional nutritional supplements,” “nutritional foods,” and “disease management.” All accessed articles published were prioritized as editorials, news articles, and WHO publications considered relevant to the review. The search scope included all the countries on the African continent. The authors conducted the literature search between June 2021 and April 2022.

## 3. Results

### 3.1. African Traditional Fermented Foods

#### 3.1.1. Cereal-Based Fermented Foods

Cereals are staple foods in Africa. The most commonly used cereals include maize (*Zea mays*), sorghum (*Sorghum bicolor*), millet (*Peninsetum americanum*), and acha or fonio (*Digitaria exilis*). These cereals are characterized by a high content of soluble non-starch polysaccharides (e.g., arabinoxylan and β–glucan), which have a health-promoting role and rich nondigestible carbohydrates (e.g., galacto- and fructooligosaccharides), excellent substrates for fermenting micro-organisms [8]. In sub-Saharan Africa, cassava (*Manihot esculenta*), also known by several regional names, is mainly a smallholder root crop, crucial for the food security of smallholder farmers, and primarily used to produce traditional fermented foods [9]. 

Fermentation of cereals is a common food processing practice performed at the household level for cereal preservation. It plays a crucial role in attaining food and nutrition security in sub-Saharan Africa [10]. Generally, fermentation positively affects the antioxidant activity of fermented grain-based foods through increased phenolic compounds, gamma-aminobutyric acid (GABA), and bioactive peptides. Changes in the vitamin contents of cereals due to fermentation vary according to the process conditions and the raw material used [11]. An increase in folate content is debated, and there is a current lack of information and research about the effect of lactic acid bacteria (LAB)-mediated fermentation on the vitamin content in fermented cereal foods [12]. Folate deficiency can cause severe deficiency during pregnancy, and there is evidence that insufficient intake of folic acid and cobalamin (vitamin B12) can drastically alter the immune system’s balance [13]. Saubade et al. (2018) [14] observed a relatively low folate content in *ben-saalga*, a pearl-millet-based fermented porridge from Burkina Faso, suggesting that folate is lost during the different processing steps. The step of spontaneous fermentation had no significant impact on folate content. Processing methods from different raw materials using corn, sorghum, and pearl millet have been shown to affect folate content and bioaccessibility in *ben-saalga* and six other African cereal-based fermented foods (*akassa*, *doncounou*, *kaffafura*, *massa*, and *ben-kida*). The main factors involved are the starting raw materials and traditional processing steps. Folate bioaccessibility is very variable and strongly influenced by the food matrix structure. Although the fermentation process positively affects the folate content in kaffa and akassa, the folate content is relatively low [15]. The optimum conditions for folate biosynthesis by LAB are still unclear, and optimizations are required to increase the LAB-mediated folate production in fermented food products [16]. Bacteria belonging to the genera *Lactobacillus*, *Lactococcus*, *Leuconostoc*, and *Pedicoccus* have been associated with cereal-based fermented foods. Other micro-organisms may be involved in fermentation processes, such as yeast species of *Saccharomyces*, *Rhodotorula*, *Candida*, *Kluyveromyces*, and *Geotrichum* genera [17], as well as filamentous molds belonging to the genera *Aspergillus*, *Rhizopus*, *Fusarium*, and *Penicillium* [8,18]. 

Pedersen et al. (2012) have identified *C. krusei* and *K. marxianus* as the dominant yeast species involved in the fermentation of *fura*, a spontaneously fermented pearl millet product consumed in West Africa. Both yeast species were capable of survival and growth under simulated gastrointestinal conditions and the transepithelial electrical resistance (TEER) of the human Caco-2 cell line, suggesting a potential probiotic property of these yeasts [19]. Owusu-Kwarteng et al. (2015) [20] obtained similar results, reporting the in vitro probiotic properties of sixteen *Lactobacillus fermentum* strains isolated from West African fermented millet dough. Interestingly, four *L. fermentum* strains showed antibacterial activity against *Listeria monocytogenes* NCTC 10527 and *Staphylococcus aureus* ATCC 1448. The yeast *Pichia kudriavzevii* strain isolated from *ogi* (a traditional, spontaneously maize-based fermented food from Benin) increased the production of folate (vitamin B9) when inoculated in pearl millet (Pennisetum glaucum)-based gruel with L. fermentum [21]. Yeast strains of *K. marxianus* and *S. cerevisiae* isolated from West Africa fermented dairy foods *lait caillé* and *nunu* and a cereal-based food *mawè* exhibited the highest probiotic potential [22]. Imade et al. (2021) [23] isolated four LAB strains identified as *Limosilactobacillus fermentum* NBRC15885, *Limosilactobacillus fermentum* CIP102980, *Companilactobacillus nantensis* LP33, and *Lactiplantibacillus garii* JCM1149 and isolated from *fufu*, *nono*, *ogi*, and *kunu* showed the ability to synthesize bacteriocin actives against pathogenic strains of *B. cereus*, *Klebsiella pneumonia*, and *S. typhimurium*. Bacteriocin is a ribosomally synthesized antimicrobial peptide that can exert a bioprotective effect against many food-spoilage and food-borne pathogenic bacteria, such as *Staphylococcus aureus*, *Listeria monocytogenes*, *Bacillus cereus*, and *Clostridium botulinum* [24].

Cereal-based fermented beverages are prevalent in Africa, and fermented food beverages are a rich source of bioactive compounds [25]. In Ethiopia, there is a long oral tradition about indigenous fermented beverages produced from different cereal raw materials, such as barley, maize, wheat, and honey. These beverages are mainly produced by acid and alcoholic fermentation (i.e., mediated by mixed cultures of micro-organisms, such as LAB and yeasts) [26,27]. *Togwa* (from maize flour, finger millet malt, Tanzania), a sweet and sour, nonalcoholic beverage, is one of the better-studied African cereal beverages. A different maize-based laboratory model of *togwa* showed how yeasts isolated from Tanzanian fermented food *togwa* can significantly increase folate content in the fermented product [28]. The yeast *Pichia kudriavzevii* isolates also showed potential probiotic abilities [21]. 

Several authors have described the potential role of food fermentation processes in reducing toxic compounds in raw food materials by harnessing autochthonous microflora involved in traditional fermentation processes or by adding selected starter cultures and adopting controlled fermentation. While few studies have documented an increase in mycotoxin levels after food fermentation processes, a decrease in mycotoxin levels has been generally reported [29]. In particular, the role of probiotics in mycotoxin biodetoxification has been described. Several micro-organisms have been reported to reduce mycotoxin accessibility, adsorption, and biotransformation in the gut through different mechanisms, including *Lactobacillus*, *Bifidobacterium*, and some *Bacillus* species of yeast *Saccharomyces cerevisiae* [30]. 

If fermentation processes seem to reduce mycotoxins levels in the final product (compared to the raw materials), traditionally processed beverages should benefit from adopting a value chain approach. Such an approach should include practical solutions to reduce mycotoxin exposure, such as educational interventions and grain-cleaning methods to optimize processing conditions/steps [31]. Recently, Nafuka et al. (2019) [32] highlighted the importance of monitoring emerging mycotoxins, aflatoxin precursors, and ergot alkaloids in sorghum malts used to produce Namibian traditional fermented sorghum-based beverages, *omalodu* and *otombo*. Indeed, the growth of mycotoxigenic fungi can be stimulated under warm, moist, and likely unhygienic conditions that may occur during the malting and milling processes. 

The LAB and yeasts can also reduce exposure to various chemical food contaminants, including metals, metalloids, and cyanotoxins [33]. The content of cyanogenic glycosides lotaustralin and linamarin is reduced in African-fermented cassava products, such as gari and fufu, during cassava fermentation by *Lactobacillus*, *Streptococcus*, and *Leuconostoc* [34]. The LAB potentially detoxify heavy metals in foods through biosorption, bioaccumulation, and transformation [35]. The detoxification of heavy metals further enhances the potential probiotic relevance of LAB, many of which are found in several Nigerian fermented foods [36].

#### 3.1.2. Meat- and Fish-Based Fermented Foods

Numerous meat-based fermented foods are also present in many traditional African diets, especially in the Mediterranean [37]. Together with dairy products, fermented meat products are also sources of peptides with antioxidant activity [11]. Recent studies have focused on the identification and quantification of bioactive peptides derived from fermented meat products and their possible roles in disease prevention [38].

Several studies have reported microbiological characterizations of fermented meat products from North Africa. For instance, Belgacem et al. (2010) [39] isolated 24 strains of *Enterococcus faecium* from *gueddid*, a traditionally Tunisian fermented meat, producing bacteriocin production with inhibitory activity against *Listeria* spp., *Enterococcus* spp., and Staphylococcus aureus. One isolate was active against *Escherichia coli* CECT 877. Nine of the antagonistic enterococci tested did not show any virulence traits or produce biogenic amines. Despite the probiotic potential of the genus *Enterococcus*, as well as their contribution to the ripening and aroma development of fermented meat products, the prevalence of virulence factors and antibiotic-resistance genes and their ability to cause disease can pose risks for food safety issues [40]. Thus, to properly evaluate food safety in traditional fermented meat productions, the monitoring of *Enterococcus* strains should be encouraged.

Boudechicha et al. (2017) [41] provided a preliminary microbiological characterization of *khliaa ezir*, Algeria’s traditional cured meat product. The LAB are the most bountiful in the product during the ripening and storage. A low level of enterobacterial population and a high general hygiene quality have been attributed to the spicing and salting thermal treatment steps. Similar results have been obtained by Benlacheheb et al. (2019) from a microbiological study on *el-guedid*, an Algerian traditional fermented red meat-based product [42]. *Aerococcus* and *Enterococcus* species isolated from *el-guedid* have exhibited a probiotic potential [43]. Bader et al. (2021) reported the results of a more comprehensive study of *el-guided* physicochemical and microbiological properties, considering the type of raw red meat and the conservation time [44]. Lactic acid bacteria and coagulase-negative staphylococci were the dominant populations in *el-guedid*, including *Leuconostoc mesenteroides*, *Lactobacillus sakei*, and *Staphylococcus saprophyticus*. In particular, *L. sakei* and *L. mesenteroides* can produce bacteriocins that could contribute to the microbiological safety of *el-guedid*. Bacteria, including LAB and coagulase-negative staphylococci, can increase the safety of fermented meat products by controlling or reducing the microbiological hazards of bacteriocin production [45]. Generally, bacteriocins have shown a tremendous inhibitory effect on *Listeria monocytogenes* in meat products [46]. Bacteriocin production could have a protective culture in fermented meats for the control or reduction in microbiological hazards; however, bacteriocins may inhibit desired starter cultures and may not be active against food spoilage bacteria [47]. 

Fermentation is also a widespread practice for fish preservation in Africa. Prominent examples are *lanhouin* (Benin and Togo), *momone*, *koobi*, *kako,* and *ewule* (Ghana), *guedj* (Gambia), *tambadiang*, and *guedj* (Senegal), *djege* and *jalan* (Mali), *fessiekh*, *kejeick*, *terkeen,* and *mindeshi* (Sudan), *dagaa* (Uganda), *gyagawere*, *adjonfa,* and *adjuevan* (Côte d’Ivoire), and *salanga* (Chad) [48]. In local cereal-based diets, fermented fish products are generally used as taste- and flavor-enhancing condiments or as a source of animal proteins. The production of traditional fish-based fermented foods is based chiefly on spontaneous fermentation processes. As for many other traditional food fermentations, such food processing techniques would require more attention both in terms of standardization of the operations and improvement in hygienic aspects [49,50,51]. The LAB and yeasts are the dominant micro-organisms in many fermented fish products [52]. Many yeast and bacterial strains have been isolated from *momoni*, a Ghanaian fermented fish condiment, with *Bacillus* species predominant [53]. However, authors have suggested that the fermentation process is mediated by the endogenous fish enzymes rather than the associated microflora due to the high pH and high salt concentrations. Farag et al. (2022) have suggested that future studies will be required to understand the better microbial impact on the quality of fermented and salted fish such as *feseekh*, *moloha*, and *renga* from Egypt [54].

*Lanhouin* is a fermented fish-based product widely used as a condiment in Benin, Togo, and Ghana. *Lanhouin* is processed by spontaneous fermentation from different fishes, such as cassava croaker/cassava fish (*Speudotolithus* sp.) or Spanish mackerel/kingfish (Scomberomorus tritor), and different processes as well. Anihouvi at al. (2007) [55] have reported changes in microbial communities during spontaneous fermentation of lanhouin from cassava fish (*Pseudotolithus* sp.). *Bacillus subtilis*, *Bacillus licheniformis*, *Staphylococcus lentus,* and *Staphylococcus xylosus* persisted up to the end of fermentation. 

Koffi-Nevry et al. (2011) [56] have studied the LAB communities in *adjuevan*, a traditional salted fermented fish (the Atlantic bumper, *Chloroscombrus chrysurus*) from Côte d’Ivoire. *Lactobacillus fermentum*, *Leuconostoc lactis*, *Pediococcus* sp., and *Streptococcus* sp. have been isolated both from the fresh fish *Chloroscombrus chrysurus* and the *adjuvant* samples. Similarly, adjuvant microbial community dynamics were produced using the whole fish and fish fillets [57]. Although the composition varied according to the preparation method, yeast, and several LAB communities were found for both. LAB included *Lactobacillus*, *Pedicoccus*, *Lactococcus*, *Streptococcus*, and Leuconostoc species, but no LAB species were dominant. Clémentine et al. (2020) [58] have instead studied yeast diversity in *adjuevan* fermentation. Seven species of yeast have been identified, and varied fermentation methods and salt concentrations used. These include *Pichia fermentans*, *Candida zeylanoides*, *Candida* sp., *Hanseniaspora osmophila*, *Kluyveromyces* sp., *Torulaspora delbrueckii*, and *Kluyveromyces marxianus*. All these yeast species have probiotic potential [59].

#### 3.1.3. Dairy Fermented Products

With its substantial social and cultural value, milk has always been a critical dietary component in sub-Saharan pastoral communities [60,61].

As for other fermented foods, fermentation was primarily used as a traditional food preservation practice for surplus milk produced during the wet season. Regarding their health-promoting properties, yogurt and fermented milk have higher antioxidant activity than milk because of the release of bioactive peptides by microbial-mediated proteolysis. Dairy products are rich sources of bioactive peptides with several activities, including immunomodulatory and antioxidant properties [38]. Several factors can influence the antioxidant power of fermented milk, such as milk origin, milk fat content, and the presence and position in milk peptides of the amino acids tryptophan, tyrosine, methionine, and fermenting micro-organism strains [62]. Increased formation of conjugated linoleic acid (CLA) and folates in fermented milk can also increase antioxidant power [11]. Dairy products are excellent sources of vitamin B12, mainly synthesized by anaerobic micro-organisms [63]. Another vitamin essential for human health is the lipid-soluble vitamin K. In particular, one of the two biologically active forms of vitamin K, vitamin K2, is predominantly of microbial origin and refers to a group of menaquinones (MKs) with different side chain lengths. Long-chain vitamin K2 forms are produced by food-grade bacteria involved in food fermentation processes, such as *Bacillus subtilis* and, interestingly, by some species and strains of LAB, such as *Lactococcus lactis* ssp. *Cremoris*, *L. lactis* ssp. *Lactis*, *Leuconostoc lactis,* and *Leuconostoc mesenteroides* [64]. Vitamin K2 can also be synthesized by bacteria belonging to the *Bacteroides* genus, one of the two most important gut microbiota genera. However, due to its poor bioavailability, the major source of functionally available vitamin K2 is the diet. Therefore, dairy products may be a predominant source of dietary MK in many regions of the world. Recently, there has been considerable interest in enhancing the MK content of dairy products through the identification and selection of MK-producing bacteria in dairy fermentations [65]. 

Fermented milk is the most widely fermented dairy product in traditional African diets. Some examples include spontaneously fermented milk, such as *iben* (Algeria, North Africa), *sussa* (Ethiopia, Somalia, Kenya, and Sudan), *ergo* (Ethiopia), *kule* and *amabere amaruranu* (Kenya), *kivuguto* (Rwanda), *amasi* (Zimbabwe), and *nunu* (Ghana). Inoculated fermentation methods, in some cases performed as semicontinuous or fed-batch fermentation processes, are also practiced. Even if not properly standardized, such inoculated fermentation methods can contribute to the stabilization of production processes. Some examples include *zabady* (Egypt), *rob*, *biruni*, *garris* (Sudan), *masse* (Mozambique), *madila* (Botswana), and *omashikwa* (Namibia). The preparation of fresh and ripened cheeses is also part of traditional diets [66].

The LAB, such as *Lactococcus lactis*, *Streptococcus infantarius* subsp. *Infantarius* and *Lactobacillus* spp. dominate African fermented dairy productions mainly as autochthonous milk microflora and indigenous microbial flora, brought by utensils and containers used for milk preservation [67]. Mesophilic bacteria (*Lactococcus* and *Leuconostoc* spp.) in fermented dairy foods are found mainly in cold climatic regions, while thermophilic bacteria (*Lactobacillus* and *Streptococcus* spp.) are found in hot climatic regions. Yeasts are often associated with fermented dairy products, e.g., *amabere amaruranu*, *gariss*, *nunu*, and *rob* [68]. Lack of standardization procedures and scarce hygiene conditions that often occur in milk production and processing can affect fermented dairy products’ shelf stability and pose risks to consumers [69,70,71,72]. A recent review about pathogenic and chemical contaminants in dairy products across sub-Saharan Africa highlighted current gaps as well as the need for robust investigations into these food safety risks [73]. 

The LAB present in dairy fermented products can exert bioprotection against food spoilage and food-borne pathogenic bacteria (e.g., *Staphylococcus aureus*, *Escherichia coli*, *Campylobacter jejuni,* and *Vibrio cholera*), mainly through a pH reduction that occurs during fermentation. Todorov (2008) [74] reported the production of bacteriocins active against *Listeria*, produced by *Lactobacillus Plantarum* isolated from *amasi* (a naturally fermented milk product from Zimbabwe). Moshba et al. (2018) have reported the production of bioactive peptides from the proteolysis of whey proteins in camel milk with antimicrobial activity against *S. aureus*, *P. aeruginosa*, *K. pneumoniae*, and *E. coli* [75]. The inhibitory effect of camel milk against pathogenic micro-organisms is well known [76]. In addition, several bioactive compounds (lactoferrin, α-lactalbumin, β-caseins and vitamin C, lysozyme, IgG and secretory IgA, and insulin-like protein) present in camel milk exhibited antioxidant, anti-inflammatory and immunomodulatory properties [77].

Rural dairy processing can also contribute to the protection of the final product against food-borne pathogenic bacteria. Two examples are the production of smoked vessels for the Ethiopian fermented milk *ergo* that can slow the growth of coliforms or the use of flavors, such as black cumin in *mish*, a ripened soft cheese from Egypt and Sudan [66]. Most artisanal cheeses in East Africa and Northern Africa are soft cheeses [78]. Some examples of fresh cheeses include *klila*, *warankasi*, *kariesh* (or *karish*), *ayib*, and *gibna*. In North Africa, hard-ripened cheeses (such as *domiati*, *mish*, and *bouhezza*) are more common than in other parts of Africa [66]. Some exceptions include *touaregh* (Mali) and *tchoukou* (Niger) [78]. Traditional cheese production at the rural level is generally characterized by scarce hygiene standards favored by a lack of cheese-making standardization in safety aspects managed empirically. Abdelfatah and Tahoun (2015) studied LAB in *Kariesh* cheese, and a variety of LAB have been isolated. In particular, *Lactobacillus rhamnosus* was the most prominent strain in *kariesh* cheese, and the high antibiotic-resistant *Lactococcus garvieae* pathogenic strain was isolated [79]. The probiotic potential of *Pediococcus acidilactici* isolated from *wara* has been recently investigated and reported [80]. *Tchoukou* cheese (Niger) is a rich source of calcium and zinc with high nutritional value [81]. A recent evaluation reported the probiotic potential of lactic acid bacteria strains isolated from *tchoukou* cheese [82]. The LAB *Lactobacillus fermentum*, *Lactobacillus intestinalis*, and *Lactobacillus acidophilus* isolated from *klila* cheese exhibited strong bactericidal activity against *S. aureus* [83].

Marino et al. 2012 [84] conducted a study to characterize healthy beneficial compounds in *bouhezza* cheese. Results showed how raw milk quality and cheese-making technology could strongly affect fat-soluble antioxidants, linolenic acid, and conjugated linoleic acid contents.

**Figure 1:**
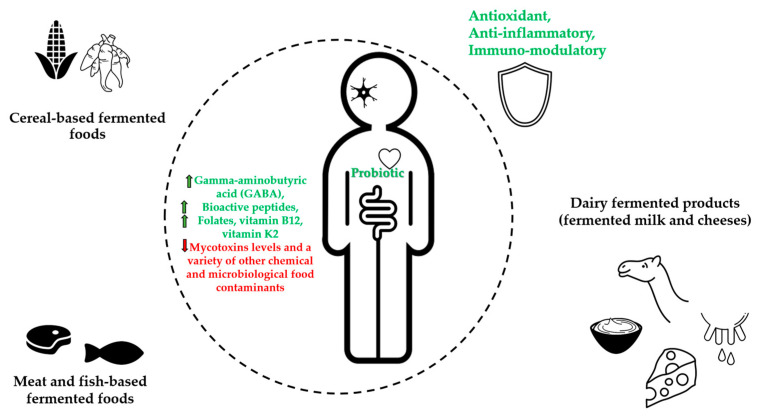
Graphical illustration of the main health effects from African traditional fermented foods.

Results about traditional fermented foods with potential health benefits are presented in Table 1 and Figure 1. 

### 3.2. Herbal Plants for Epidemics and Pandemics

In addition, nine herbal remedies and spices belonging to eight plant families have antioxidant, immunomodulatory, anti-inflammatory, neuroprotective, hepatoprotective, and cardioprotective abilities, with some having antiviral properties. Peptides, flavonoids, alkaloids, sterols, ascorbic acid, minerals, vitamins, and saponins are some of the bioactive compounds in herbal remedies. 

#### 3.2.1. *Garcinia kola* Heckel (Fam. Clusiaceae) [Bitter Cola]

Every part of bitter kola (*Garcinia kola* Heckel) is traditionally used in Africa to treat typhoid fever, bronchitis, bacterial infections, malignant tumors, skin infections, tuberculosis, gastritis, colds, and jaundice [85,86]. The plant is indigenous to Africa and pharmacologically evaluated in animal studies to have antiviral, antiasthma, antioxidant, antidiabetic, antihypertensive, antibacterial, antiasthma, and hepatoprotective activities [87,88,89]. Although *G. kola* has not been scientifically documented for use in pandemics, its traditional usage and bioactive constituents make the plant a candidate for use in pandemics and epidemics in Africa [85]. For instance, in recent reviews, *G. kola* has been suggested as a potential and promising medicinal plant for the treatment of coronaviruses due to its antiviral and antioxidant activities [90,91,92]. Similarly, *G. kola*, the active constituent of *G. kola*, has been reported to be active against polioviruses, measles virus, yellow fever virus, influenza, and herpes simplex virus-1 [93,94]. *G. kola’s* immunorestorative and immunomodulatory activities make it a significant plant in diseases causing immunodeficiencies, such as COVID-19 and AIDS [95]. The bioactive compounds found in *G. kola* include alkaloids, phenols, saponins, sterols, tannins, garciniflavanone, kolanone, garcinoic acid, kolaflavanone, and kolaviron [85,89,96]. The seed is also rich in phosphorous and potassium [96]. Kolavoviron has significant anti-inflammatory and antioxidant activity. It was reported to be effective against viruses via immunomodulating activities, metal chelating, and as a potent radical scavenger [93,97,98]. Furthermore, kolavoviron modulate oxidative stress via stimulation of phase 2 detoxification of enzymes, proving its chemopreventive effects. This action mitigates the expression of COX-2 and iNOS at the molecular level, downregulating AP-1 DNA and NF-kB binding, and mitigating oxidative damage to biomolecules [97,99,100]. Other derivatives of *G. kola*, such as guttiferones and garcinol, have been reported to inhibit the cytopathic effects of HIV [101]. Toxicological studies in rats and mice showed *G. kola* to be safe [102]. The long history of the seed’s usage and the safe toxicological profile make *G. kola* a suitable candidate for clinical trials in pandemics.

#### 3.2.2. *Artemisia afra* Jacq. (Fam. Asteraceae) [African Wormwood]

Dihydroxybishopsolicepolide, scopoletin, acacetin, flavonoids, and yomogiartemin are the common phytochemicals identified and described in *Artemisia* [103,104]. The shrub is abundant in Northern, Eastern, and Southern Africa. It has been reported for use traditionally in treating influenza, respiratory infections, cough, malaria, diabetes, and fever [105]. The plant’s bioactive ingredients have been reported to have potent antioxidant activity through scavenging hydrogen peroxide and hydroxyl ions and modulating reactive oxygen species, thus, making *Artemisia* a protective agent that strengthens the antioxidant defense mechanism [106]. Furthermore, the phytochemicals in Artemisia and their derivatives provided selective cytotoxicity in randomized double-blinded subjects treated for colorectal cancer [107]. Slezakova and Ruda-Kucerova (2017) [108] further reported *Artemisia’s* promising potential in hepatocellular carcinoma, lung cancer, and breast cancer. 

*Artemisia* also has significant antiviral activities against influenza virus A, human herpes viruses 1 and 2, hepatitis B and C, and HIV-1 viruses [109,110,111]. *Artemisia’s* general mechanism of action blocks the host-cell–type and metabolic requirements for viral replication by inhibiting the central regulatory activity of viral-infected cells [109,111,112].

*Artemisia* is an African indigenous traditional medicinal plant for treating diseases associated with pandemics and epidemics. Its antioxidant, anti-inflammatory, antiviral activities, low toxicity, and safety make *Artemisia* a potential drug candidate for the prevention and treatment of diseases in pandemics and epidemics on the continent of Africa [105,113].

#### 3.2.3. *Piper guineense* (Fam. Piperaceae) [African Black Pepper]

One of the most valuable African plant species widely applied in traditional medicine is *P. guineense*. The plant has potent antioxidant properties with strong antibacterial, anticancer, and antiviral properties [114]. An ethnopharmacological survey showed that the plant is used in traditional African settings for sexually transmitted diseases [115]. Piperine, piperlongumine, ligans, monoterpenes, terpenoids, sterols, sesquiterpenes, and volatile oils are some of the bioactive compounds in *P. guineense* [116]. These alkaloids are scaffolds for discovering new drugs since they contain antimicrobial pharmacological properties [117,118]. For instance, piperine, a potent antibacterial agent, inhibits the efflux pump in *Staphyloccocus aureus*, making it a potential phytochemical for multidrug-resistant bacteria [119].

Furthermore, a recent study suggested that a combination of piperine and rifampicin improved rifampicin’s effectiveness, making piperine an agent that can reduce the adverse effects of rifampin when used in clinical therapy [120]. Apart from piper’s reported effectiveness as an anti-inflammatory and antiproliferative, the bioactive compound was also reported to demonstrate protection against chronic diseases based on clinical studies [110]. Furthermore, piperlongumine is effective as an anticancer, antifungal, antihelminth, and in treating many neglected tropical diseases in Africa, making *P. guineense* a valuable plant used in pandemics [110]. The effectiveness of piperlongumine in neurodegenerative diseases is due to its ability to inhibit or reduce the synthesis of prostaglandins E_2_, nitric oxide, cyclooxygenases-2, nuclear factor kappa B, interleukin-6, and tumor necrosis factor-alpha 9 [121,122]. Osho et al. (2016) reported the antiviral activity of *P. guineense’s* methanolic extract in broiler chickens infected with Newcastle disease virus (NDV) [123]. The numerous underlying mechanisms of action and multitargeting potentials of *P. guineense* are still being studied, even though the benefits have existed in Africa for a long time [122].

#### 3.2.4. *Achyranthes Aspera* Linn. (Fam. Amaranthaceae)

The origin of *Achyranthes aspera* is in Africa, even though some literature indicates that the plant is also to be native to South Asia [124]. The plant has been associated with its usefulness in pandemics due to its significant antioxidant and immune-boosting abilities and its antiviral effects [124,125]. The antiviral potential of a methanolic extract of *Achyranthes aspera* was evaluated against herpes simplex virus type 1 and type 2. *A. aspera* demonstrated good anti-*Herpes simplex* virus activity [125]. In addition to its usefulness in pandemics, the entire part of *A. aspera* has been traditionally used in Africa and other parts of Asia for dysentery, arthritis, malaria, hemorrhoids, fever, pain, and diarrhea [124]. In addition, the plant is diuretic, anti-inflammatory, antiasthmatic, and valuable for pneumonia [126,127,128]. 

Triacontanol, eugenol, ecdysterone, and betaine are the main bioactive compounds in *A. aspera*. Furthermore, the plant’s antioxidant and immune-boosting activities are enhanced by ascorbic acid [128,129,130,131]. The ascorbic acid in *A. aspera* significantly boosts immunity and alleviates inflammation in the SARS-coronavirus by conferring antioxidant properties [92]. The chemopreventive effects of *A. aspera* have also been reported in a few studies. Saponin fractions of *A. aspera* considerably reduce early antigen activation elicited by the tumor promoter 12-O-tetradecanoylphorbol-13-acetate in Raji cells by Epstein-Barr virus. [132]. Furthermore, *A. aspera* induced apoptosis through a mitochondrial-mediated pathway [133].

#### 3.2.5. *Allium sativum* L. (Fam. Liliaceae) [Garlic]

Garlic (*Allium sativum* L., Fam. Liliaceae), though historically believed to originate from West China, is presently cultivated and used globally as a spice, immune booster, and a remedy during pandemics. Garlic is the first remedy to prevent and treat pandemics, such as influenza, typhus, cholera, and dysentery [134]. Garlic was fed to pyramid builders to boost immunity and was reported as a nutritional supplement in ancient inscriptions in Egyptian pyramid plates. In addition, garlic provided the builders with vitamins, balance, and the energy to pull the heavy plates used in building the pyramids [135].

Traditionally, garlic has been used in Africa to manage bacterial, parasitic, viral, and other infectious diseases [136,137,138]. Other uses included gynecological diseases, toothaches, snake bites, arthritis, and hypertension [136,137,139,140]. In general, garlic extracts are effective as antioxidants, anticancer, and antimicrobials and tend to reduce the risk of cardiovascular events [134,141,142]. Experimental animal and clinical studies have provided evidence of garlic’s effectiveness in managing the common flu, diabetes, hypertension, arthritis, and cancer prevention [142,143].

Over a hundred bioactive compounds are found in garlic, with allicin (thiosulfate) being the most active and responsible for garlic taste and smell [144]. Other sulfur-containing bioactive substances found in garlic include diallyl trisulfate, ajoenes, diallyl disulfide (sulfides), 2-vinyl-(4H)-1,3-dithiin, 3-vinyl-(4H)-1,2-dithiin (vinyldithiins), and alliin, which, constitutes majorly cysteine sulfoxide [145,146,147]. 

Garlic is used in pandemics due to its antioxidant, antiviral, and antibacterial activities [88]. Reduction in the synthesis of oxygen-free radical species has been reported with garlic when it is frequently consumed, promoting antioxidant activity [147,148]. Similarly, garlic extracts have been reported to reduce glutathione peroxides and superoxide dismutase in rats’ hepatic tissues [149,150]. In addition, high radical scavenging activity with different sulfur-containing substances in garlic, phenols, and flavonoids has been demonstrated with garlic extracts [151]. Other antioxidant activities of the bioactive compounds in garlic include a decrease in the synthesis of reactive nitrogen and oxygen species by diallyl sulfide through the enzymatic suppression of cytochrome P450-2E1, leading to hepatoprotection [147,152]; inhibition and H_2_O_2_-induced DNA damage with saponins extracted from garlic [153]; and the inhibition of NADPH oxidase 1 through the prevention of reactive oxygen species with alliin [154]. 

In a recent review, Batiha et al. (2020) [147] reported that garlic extract is effective against diverse viruses, such as vesicular stomatitis virus, human rhinovirus type 2, influenza virus type 3, human cytomegalovirus, influenza B type virus, and herpes simplex 1 and 2 [155]. The inhibition of adhesive interaction and fusion of leukocytes with ajoenes; inhibiting the synthesis of thiol enzymes by allicin; and the enhancement of natural killer-cell activity through the destruction of cells infected by viruses’ diallyl trisulfide on human cytomegalovirus are a few mechanisms of garlic’s action [147,155]. Finally, allicin has a wide range of activities against many bacterial organisms, including *K. aerogenes*, *E. faecalis*, *S. enterica*, *E. coli*, *S. pyogenes*, *S. mutans*, *Mycobacteria*, *Shigella*, *P. vulgaris*, and *P. aeruginosa* [137,149,156,157,158,159]. The ability of garlic to inhibit NF-kB and modulate cytokine expression makes it an immunomodulatory and effective plant for pandemics in Africa. Finally, prostaglandin-E2, COX-2, and nitric oxide production inhibition lead to a significant reduction in the synthesis of inflammatory interferon γ, interleukin-6, and TNF- α with garlic [159]. 

#### 3.2.6. *Moringa oleifera* Lam. (Fam. Moringaceae)

*Moringa oleifera* is commonly called the miracle tree in Africa because of its numerous traditional and pharmacological activities against various diseases. The plant’s multipurpose nutritional and health benefits have been widely reported in Africa and most parts of the world [140,160,161,162]. The plant has been traditionally used in Africa for food, livestock feed, nutrition, and medicine [163]. Recently, *Moringa* has been used in biofuel production, cosmetics, and water purification [161,164]. The phytochemicals responsible for *Moringa*’s biological effects include isothiocyanate, phenolic acids, polyphenols, sterols, alkaloids, terpenes, flavonoids, and flavanol glycosides [160,161,162,163]. Some ethnopharmacological activities of moringa include antioxidant, parasitic diseases, antituberculosis, anticancer, antidiabetic, anti-inflammatory, sexually transmitted infections, typhoid fever, cardioprotective, neuroprotective, antihypertensive, and hepatoprotective effects [162]. Even though moringa significantly decreased triglyceride, cholesterol, and glucose levels in rats; the plant is safe and has a high therapeutic index [165]. *Moringa* leaves are rich in beta carotene, minerals, and proteins, essential compounds lacking in most populations found in developing nations. The ability of *Moringa* to boost the immune system and help the body fight infections has been reported. The plant showed some significant activities against various viruses, including HIV, and has been reported to be used in managing AIDS and diseases related to AIDS infections [166,167]. In addition, *Moringa* is effective against the influenza A virus, new castle disease virus, herpes simplex virus, Epstein-Barr virus, hepatitis B virus, and foot-and-mouth disease virus in cloven-footed animals [168]. Protection of infected host cells from cytopathic effect; decrease in the levels of cytokines; inhibition of the expression and nuclear transfer of cellular proteins transcription factor EB leading to a weakening of the autophagy infected cells; and the inhibition of viral replication in host cells are some of the reported antiviral mechanisms of action of moringa [169]. These biologic activities made *Moringa* an African medicinal plant used to prevent and treat pandemics and diseases related to pandemics in the African continent. 

#### 3.2.7. *Zingiber officinale* R. (Fam Zingiberaceae) [Ginger]

*Zingiber officinale* R. (Fam Zingiberaceae) is presently widely cultivated in most African countries for the prevention and treatment of common influenza, cough, sore throats, arthritis, lung diseases, peptic ulcer disease, hypertension, and infectious diseases, such as bacterial and viral infections. Although Ginger was initially used as a food spice and for medicinal plants in China, the use of ginger in Africa for the prevention and treatment of diseases has been documented [170]. Presently over 100 species of ginger are cultivated worldwide; however, *Z. officinale* is the most cultivated and used as an ingredient for food and medicinal plants [171,172]. 

The US Department of Agriculture (2013) identified steroids, phenols, and alkaloids as the bioactive compounds found in ginger with therapeutic activities. Other active compounds include gingerols, zingerone, zingiberol, paradols, and shogaols [173,174,175,176]. Ginger has been widely reported to have antiarthritic, anticancer, antioxidant, anti-rhinoviral, antimicrobial, and antiglycemic pharmacological activities [174,177,178]. Shogaol and gingerol have significant pharmacological activity compared to other bioactive compounds found in ginger [179]. 

Ginger’s antiviral, antioxidant, and antibacterial effects make it a viable medicinal herb and spice used to prevent and manage pandemics in Africa [180]. Chang et al. (2013) demonstrated that fresh ginger acts by blocking viral attachments and internalization and is effective against human respiratory syncytial virus-induced plaque formation on airway epithelium [181]. Similarly, Rathinavel et al. (2020) reported that 6-gingerol, a phytocompound from *Z. officinale*, showed high binding affinity against SARS COVID-19, including spike proteins, RNA binding, and viral proteases proving antiviral activity, making 6-gingerol a promising bioactive compound for COVID-19. Good ADME pharmacokinetic properties and excellent drug-likeness parameters with zero rule violations were demonstrated by 6-gingerol [182]. Generally, the Zingiberaceae plant family (*Zingiber officinale*, *Curcuma longa*, and *Aframomum melegueta*) is effective against a wide variety of viruses, such as *Enterovirus* 71, Japanese encephalitis virus, Epstein-Barr virus, *Herpes simplex* virus types 1 and 2, influenza A virus, human immunodeficiency viruses, coronavirus SARS-CoV-1, rhinovirus, chikungunya virus, and respiratory syncytial virus. In addition, these herbs are significant immune system boosters and excellent sources of nutrition [180].

Furthermore, besides possessing antiviral activities, *Z. officinale* has significant antioxidant activities. Zingerone, for instance, suppresses lipid peroxidation and scavenges hydroxyl ions and peroxides [183,184,185]. Similarly, 6-gingerol effectively protects against ultraviolet B-induced skin disorders in rats by inhibiting the induction of NF-kappa translocation, proteins, and cyclooxygenase-2 mRNA [186]. *Z. officinale* terpenoids significantly rendered endometrial cancer cells ineffective by promoting the stimulation of p53 [178]. In addition, the combination of 10-gingerol, 8-gingerol, 6-gingerol, and 6-shogaol prevented the rapid multiplication of PC-3 prostate cancer cells, thereby providing cytoprotective effects [186,187]. Other studies on ginger’s cytoprotective effects reported ginger powder suppressing the synthesis of the COX-1 enzyme associated with intestinal cancers [188]. Similarly, a decrease in the synthesis of metalloproteinase-9 and the colonization of breast cancer cells was demonstrated by 6-shogaol [189]. In addition, 6-gingerol stimulates the generation and formation of new blood vessels, aiding in the prevention of the spread of cancer cells [190]. Preventing the synthesis of prostaglandin synthase or 5-lipoxygenase with gingerol and shogaol effectively inhibits the expression of prostaglandins and leukotrienes [191,192]. Although the rhizome is commonly used, all the parts of ginger are utilized in African traditional medicine [92].

#### 3.2.8. *Momordica charantia* (Fam. Cucurbitaceae) [Bitter Melon]

Bitter melon (*Momordica charantia*) has been used as a medicinal plant to treat viral and gastrointestinal infections and for ritual purposes in Africa [193]. Bitter melon has been traditionally used to treat diabetes [194]. It contains several bioactive compounds that contribute to its antiviral and antioxidant activities [193,195,196]. The fruit contains minerals, vitamins, phenolic compounds, triterpenes, lipids, proteins, glycosides, steroids, saponins, and flavonoids [195].

*M. charantia* possessed antiviral activities and was reported to be effective against HIV-1, dengue virus, and hepatitis B [197]. Rebultan (1995) reported that *M. charantia* significantly increased and normalized CD4 count and CD4/CD8 ratio in HIV-infected persons while reducing recurrent respiratory infections [198]. 

In addition, *M. charantia* has been suggested as a promising anticancer agent whose bioactive compounds target cancer cells and are used to correct metabolic aberrations [199]. Autophagy and apoptosis are the mechanisms of action of *M. charantia*-mediated cell death in colon and breast cancers, respectively [200,201]. The plant’s potential medicinal effects and applications for HIV, diabetes, and cancers made it a good candidate for the treatment of pandemic-related viral diseases and to boost immunity in poor African populations where sophisticated healthcare systems are not available [194,197,200,202]. The variety of steroids and protein compounds isolated from *M. charantia*, such as kuguacin C and kuguacin E, give the plant its antiviral properties. In a recent review, Jia et al. (2017) summarized the antiviral activity of the Momordica anti-HIV protein of 30 kD (MAP30). The extracted protein from *M. charantia* inhibits HIV-1 DNA replication in monocytes and selectively kills lymphocytes and macrophage-infected with HIV with minimal toxicity to the cells unaffected by HIV [196]. With a rich collection of phytochemicals and years of successful application in managing and preventing various diseases, *M. charantia* is a potential source of relief in managing Africa’s present and future epidemics and pandemics.

#### 3.2.9. *Curcuma longa* L. (Fam. Zingiberaceae) [Tumeric] 

Several African scientists have reported the use of *C. longa* in African traditional folk medicine and its use as a food spice [203,204,205,206]. Although the plant is cultivated throughout the entire continent of Africa and other parts of the world, Western and Eastern Africa seem to have wider plant cultivation than the rest of Africa [207]. C. *longa* has been traditionally reported for use in conjunctivitis, smallpox, and sinusitis [205,206,208]. The plant has antioxidant, anticancer, immunomodulator, and antimicrobial properties [206,208]. These pharmacological potentials and properties of *C. longa* have been exploited in Africa for pandemics and epidemics [208]. Curcuminoids, quercetin, curcumin, zingiberine, borneol, alpha-phellandrene, and a variety of sesquiterpenes are some of the bioactive phytochemicals found in *C*. *longa* [204,206,208]. Aggarwal et al. (2016) reported curcumin as a potent antioxidant by suppressing NF-κB and NF-α, in addition to its antibacterial effect on organisms, such as *Vibrio cholera* and *Klebsiella pneumonia* [208]. Furthermore, African *C. longa* possessed substantial antiviral activities making it a potential plant in pandemics associated with viruses. The plant is effective against HIV-1, H1N1, hepatitis C, parainfluenza virus type 3, H6N1, human papillomavirus, and coxsackievirus B3 [204,206,208]. The ability of curcumin to inhibit viral hemagglutination, suppress viral replication, and down-regulate viral transcription made curcumin a candidate for use in pandemics [208]. The antibacterial, antiviral, anti-inflammatory, and antioxidant activities of *C. longa* made the plant a valuable and potential therapy in pandemics throughout Africa [209].

**Figure 2:**
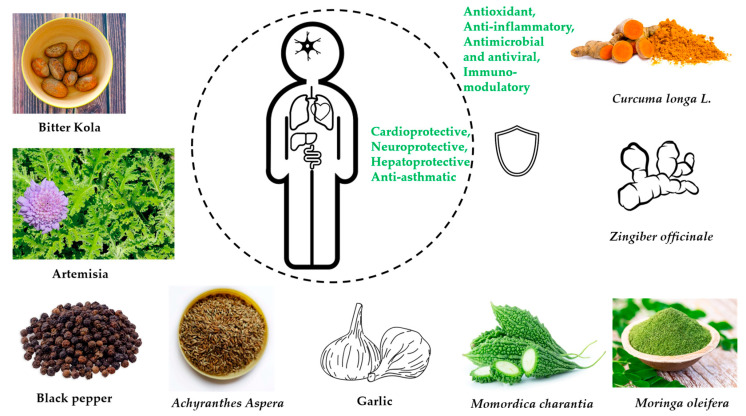
Graphical illustration of main health effects from traditional herbal remedies.

The bioactive compounds in medicinal plants, their pharmacologic activity, and their potential to mitigate infectious diseases during epidemics and pandemics are presented in Table 2 and Figure 2.

## 4. Discussion

Fermentation processes are effective food preservation methods and valuable transformation processes that strongly affect human nutrition and diet globally. Other than improving food digestibility and palatability (development of food texture, flavor, and aroma), fermentation can reduce or eliminate toxic compounds and produce bioactive substances that can exert a protective action against undesirable micro-organisms, including pathogens [211]. In addition, compared to raw food materials, fermented foods possess an increased dietary value (e.g., linked to the production of micronutrients) with potential positive impacts on consumers’ health. Lactic acid bacteria are a group of gram-positive bacteria mainly involved in food and feed fermentation and generally associated with healthy gut mucosa in animals and humans. LAB is prominent in all traditional fermentations except for alcoholic fermentation (created by yeasts). If administered by diet, both LAB and some yeast species have exhibited potential probiotic effects associated with beneficial health effects for humans and animals. These effects mainly rely on probiotics’ ability to host gut colonization and the maintenance of suitable intestinal microflora homeostasis, which is central for many health-related aspects, including metabolic, and neurobehavioral immunomodulation [212,213]. Although the multiple mechanisms involved have not been fully elucidated, probiotics can boost both innate and acquired immune systems [214]. Thus, probiotics can potentially be active in preventing or treating infectious diseases in the gastrointestinal and upper respiratory tracts [215]. Foods and fermented products containing probiotics showed significant potential effects on preventing and treating viral diseases, including COVID-19 [216,217]. Recently, the potential role of vitamin K deficiency as a risk factor for the severity of COVID-19 has been investigated. In particular, due to its longer half-life, and greater extrahepatic potential (compared to the K1 form), vitamin K2, of which dairy foods are a significant dietary source [65], could play a prominent role. Vitamin K2 has a key role in bone formation, blood coagulation, inhibition of calcification in arteries, and thus, in cardiovascular health. Some premorbid conditions, such as hypertension, diabetes, cardiovascular diseases, and obesity, seem to increase the morbidity and mortality of COVID-19. Janssen et al. (2021) made the observation that such disorders are associated with elastic fiber pathologies as well as vitamin K insufficiency. They, therefore, hypothesized that vitamin K could represent the missing link between pulmonary damage and thrombogenicity [218]. Linneberg et al. (2021) have recently tested this hypothesis. Results showed that vitamin K status was markedly lower in hospitalized COVID-19 patients compared to population controls and that low vitamin K status was associated with mortality in patients with COVID-19 in age- and sex-adjusted analyses. These findings suggest that vitamin K could play a role in the disease mechanisms in COVID-19. However, comorbidities could be part of the causal pathway or confounders of the association of vitamin K status with mortality. Whether vitamin K supplementation in COVID-19 patients can change the course of the disease and prevent death or long-term consequences of COVID-19 remains to be tested in randomized clinical trials [219].

To properly evaluate the health risks and benefits associated with consuming traditional fermented foods, it is pivotal to acquire deeper knowledge about the complex microbial ecology and dynamics behind traditional fermentation processes. For instance, further research are required to better characterize key aspects, such as the production of long-chain vitamin K2 in LAB, the interactions of the vitamin K2-producing bacteria, and the total amounts of the different MKs in fermented foods [220]. These studies should also include a complete characterization of natural pro-technological microbial communities colonizing raw food materials and spoilage, and harmful micro-organisms contaminating fermented foods. Nowadays, omics approaches, such as phylobiomics, metagenomics, and metatranscriptomics, are entirely changing the study of microbial food ecology, providing new insights into microbial consortia and their dynamics in fermented foods [221].

Traditional fermented foods constitute a significant component of African diets, including several edible products starting from a wide range of raw food materials, such as cereals and other grains, milk, meat, and fish. Traditional fermentation methods rely on spontaneous fermentation and inoculated fermentations. Spontaneous fermentations involve the action of both endogenous enzymes and micro-organisms naturally present in food raw materials under water activity, temperature, and time conditions. Spontaneous fermentation processes cannot be fully controlled or predicted because they depend on complex and heterogeneous microbial communities. This condition can lead to slowed or even failed fermentation processes and variations in fermented food quality. Inoculated fermentation methods are based on the addition of a small portion of fermented food to the raw food material to be fermented. This process can stabilize the composition of fermenting microbial communities and accelerate fermentation rates. African fermented foods and beverages are produced using different types of fermentation: lactic acid and alcoholic fermentation are the most diffuse, followed by acetic acid and alkaline fermentation [47]. 

On the other hand, humans have used herbal remedies since ancient times. Due to Africa’s rich flora and fauna, the continent has extensively used medicinal plants for various ailments, even before scientific discoveries [204]. Medicinal plants still play a vital role in treating any disease in Africa, and in many communities, they are the primary healthcare or integrated into the healthcare system [222,223]. The World Health Organization reported that as of 2020, more than 34 research institutes on the African continent are dedicated to indigenous medicinal research, with approximately forty countries having traditional medicine policies [224]. The COVID-19 pandemic further highlighted the traditional and herbal remedies used in Africa to tackle epidemics and their use in alleviating or managing symptoms associated with pandemics [225,226]. 

In addition to the variety of foods and medicinal plants found on the African continent that play an essential role in providing community resilience against infectious diseases during epidemics and pandemics, foods from other continents have also been reported to provide immunity against epidemics and pandemics. For instance, Chinese herbal medicines have also been reported to help prevent and manage viral diseases [227]. For instance, *Astragulus membranaceus* treats common upper respiratory infections and the common cold [228]. Ginseng root is valuable in preventing viral respiratory infections, such as those due to strains of influenza [229,230]. At the same time, *Pelargonium sidoides* is an efficacious herbal therapy for inhibiting respiratory viruses’ replication [230].

Additionally, documented evidence about preventing SARS and H1N1 influenza in high-risk people implies that Chinese herbal remedies could provide an alternative strategy as immune boosters in the present and future epidemics and pandemics [228]. Other traditional Chinese bioactive food compounds, such as kaempferol, quercetic, flavonoids from litchi seeds, and phenolic compounds, have been shown to inhibit SARS 3-chymotrypsin-like protease (3CLpro) enzymatic activity. Additionally, the 3CLpro enzyme is essential for SARS-CoV replication and has been suggested as a potential agent for SARS-CoV prevention and supportive care for COVID-19 patients [231,232].

Other than Chinese herbal remedies, the Mediterranean diet (MD) provides optimal nutritional quality associated with diverse anti-inflammatory and metabolic properties and immunocompetence [233]. The traditional Mediterranean diet is plant-based and rich in fresh vegetables, fruits, nuts, whole grains, olive oil, and fish. Greene et al. [234] showed an inverse association between compliance with the Mediterranean diet and COVID-19 pandemic cases and deaths in some European countries. 

The MD is rich in fibers, folates, selenium, β-carotene, flavonoids, omega-3 fatty acids, mono-unsaturated fatty acids, and vitamins C, and E. The inverse association between MD and COVID-19 is attributed to the optimal nutritional quality and the anti-inflammatory profile of Mediterranean diets, thereby conferring immune support for COVID-19 [234].

Furthermore, the overall well-being associated with MD adherence is attributed to a decrease in the risk of common COVID-19 comorbidities, such as cancer, cerebrovascular disease, chronic kidney disease, chronic obstructive pulmonary disease, diabetes type 2, cardiovascular disease, hypertension, and obesity [225,226,227,228,229,230,231,232,233,234,235,236,237]. The rich fiber in a Mediterranean diet modulates nutrient satiety and absorption, which contributes to healthy weight maintenance. Undoubtedly, the significant role of plant-based diets and nutrition in supporting the immune response and providing optimal nutritional quality for immunocompetence, whether in Africa, Asia, or the Mediterranean, is due primarily to the bioactive compounds found in the plants. Hence the need to explore bioactive plant-based therapies for future epidemics and pandemics.

Medicinal plants have a wide range of chemical diversity. They have contributed significantly to drug discovery due to their minimal adverse effects, safety, efficacy, availability, accessibility, and general acceptability within most communities, especially in Africa. The ability of medicinal plants to exhibit antioxidant, antiviral, antidiabetic, anti-inflammatory, anticancer, and immunomodulatory properties has been well documented [238,239]. The bioactive compounds found in medicinal plants give them a broad spectrum of pharmacological and therapeutic activities. Phenolics, organosulfur compounds, flavonoids, coumarins, terpenoids, alkaloids, and steroids are some of the bioactive substances found in medicinal plants that accord their pharmacotherapeutic benefits [240]. For instance, bioactive peptides found in plants have been reported to have little or no toxicity, do not accumulate in the body, and are very effective even at low concentrations. In a recent review, Akbarian et al. [241] summarized the applications of bioactive peptides as antihypertensive, antioxidants, antithrombotic, antimicrobial, antiaging, metal chelating, and in lowering cholesterol [241]. Similarly, antioxidants in foods and medicinal plants possess anticancer, anti-inflammatory, antiaging, and immunomodulatory properties [238]. 

## 5. Conclusions

Even though personal and public hygiene and vaccinations are the most recommended methods to curb the spread of infections, natural micronutrients in food and herbal medicines can help boost immunity and provide community resilience against infectious organisms in epidemics and pandemics. The diversity of foods and medicinal plants on the African continent could have contributed to the lower rates of COVID-19 deaths reported in the region and other equally essential factors reported by other authors. Bioactive compounds in food and plants significantly support the immune system and help to increase resistance against infectious diseases. The unique foods and medicinal plants found on the African continent could play an essential role in providing community resilience against infectious diseases during epidemics and pandemics. There is a need to investigate nutritional, herbal, and traditional practices in epidemics and pandemics that support healthy living and longevity within the African continent. 

## Figures and Tables

**Table 1:** Traditional African fermented foods with potential health benefits, including the strength of the immune system.

Fermented Foods	Raw Food Materials	Micro-Organisms	Bioactive Compounds	Potential Health Benefits	References
Cereal-based fermented foods	Maize (*Zea mays*), Sorghum (*Sorghum bicolor*), Millet (*Peninsetum americanum*), Acha or Fonio (*Digitaria exilis*), Cassava (*Manihot esculenta*)	Bacteria (*Lactobacillus*, *Lactococcus*, *Leuconostoc Pedicoccus* genera) Yeasts (*Saccharomyces*, *Rhodotorula*, *Candida*, *Kluyveromyces,* and *Geotrichum* genera) Filamentous molds (*Aspergillus*, *Rhizopus*, *Fusarium,* and *Penicillium*, genera)	Soluble non-starch polysaccharides (e.g., arabinoxylan and β–glucan) Nondigestible carbohydrates (e.g., galacto- and fructo–oligosaccharides) Folates	Promote rich nondigestible carbohydrates (prebiotics), increase in phenolic compounds, gamma-aminobutyric acid (GABA), and bioactive peptides contents. Increase folates, decrease mycotoxins levels, increase health benefits of probiotic consumption, reduce exposure to a variety of other chemical food contaminants and detoxification	[8,17,18,29, 33,35,36]
Meat- and fish-based fermented foods	Meat, Fish	Bacteria (*Leuconostoc, Lactobacillus*, *Enterococcus* *Aerococcus*, *Bacillus* genera) Yeasts (*Pichia*, *Candida*, *Hanseniaspora*, *Kluyveromyces Torulaspora,* and *Kluyveromyces* genera)	Bioactive peptides, Bacteriocins	Antioxidant activity, increase health benefits of probiotic consumption, reduction of microbiological hazards	[11,38,45, 52,59]
Dairy fermented products (fermented milk and cheeses)	Milk	Bacteria (*Lactococcus Leuconostoc Streptococcus, Lactobacillus, Pediococcus* genera) Yeasts (*Saccharomyces*, *Candida*, *Kluyveromyces* genera)	Bioactive peptides, Conjugated Linoleic Acid, Vitamin B12, Vitamin K2 Bacteriocins	Antioxidant, immunomodulatory, source of vitamin B12 and vitamin K2, increase health benefits of probiotic consumption, protection against food-spoilage	[11,38,61,65 68]

**Table 2:** African herbs/medicinal plants with immune-boosting and antiviral potentials.

Herbs/ Medicinal Plants	Traditional Uses	Bioactive Substances	Pharmacologic Activity	Immunologic Activity	Antiviral Activity	References
*Garcinia kola* Heckel (Fam. *Clusiaceae*) [Bitter cola]	Typhoid fever, bronchitis, bacterial infections, malignant tumors, skin infections, tuberculosis, gastritis, cold, jaundice	Alkaloids, phenols, saponins, sterols, tannins, garciniflavanone, kolanone, garcinoic acid, kolaflavanone, and kolaviron	Antiviral, antiasthma, antioxidant, antidiabetic, antihypertensive, antibacterial, antiasthma, and for hepatoprotective activities	Antioxidant, hepatoprotective, immunomodulatory, metal chelating, potent radical scavenger, modulate oxidative stress	Polioviruses, measles virus, yellow fever virus, influenza, herpes simplex Virus-1, HIV	[85,86,93,94,97,99,201]
*Artemisia* Afra Jacq. (Fam. *Asteraceae*)	Influenza, respiratory infections, cough, malaria, diabetes, and fever	Dihydroxybishopsolicepolide, scopoletin, acacetin, flavonoids, yomogiartemin	Cytotoxic, anticancer, antiviral	Antioxidant, anti-inflammatory	Influenza virus A, human herpes viruses 1 and 2, Hepatitis B and C, HIV-1 viruses	[103,104,105,106,109,110,113]
*Piper guineense* (Fam. *Piperaceae*) [African black pepper]	Sexually transmitted diseases	Piperine, piperlongumine, ligans, monoterpenes, terpenoids, sterols, sesquiterpenes, and volatile oils	Antibacterial, anticancer, antiviral, antiproliferative, antifungal, antihelminth	Antioxidant, anti-inflammatory	Newcastle disease virus	[103,114,120,122]
*Achyranthes Aspera* Linn. (Fam. Amaranthaceae)	Dysentery, arthritis, malaria, hemorrhoids, fever, pain, and diarrhea	Triacontanol, eugenol, ecdysterone, betaine, ascorbic acid	Diuretic, anti-inflammatory, anti-asthmatic, and valuable for pneumonia	Antioxidant, immune boosting, chemopreventative	Herpes simplex virus type 1 (HSV-1, oral herpes) and type 2 (HSV-2, genital herpes).	[92,124,125,126]
*Allium sativum L.* (Fam. Liliaceae) [Garlic]	Influenza, typhus, cholera, dysentery, toothaches, snake bites, arthritis, and hypertension	Phenols, flavonoids, saponins, allicin (thiosulfate), diallyl trisulfate, ajoenes, diallyl disulfide	Anticancer, antimicrobial, flu, diabetes, hypertension, arthritis, and for the prevention of cancer	Antioxidants, immune booster	Vesicular stomatitis virus, Human rhinovirus type 2, influenza virus type 3, human cytomegalovirus, influenza B type virus, and herpes simplex 1 and 2	[134,142,143,147,155,210]
*Moringa oleifera* Lam. (Fam. Moringaceae)	Food, livestock feed, nutrition, medicines	isothiocyanate, phenolic acids, polyphenols, sterols, alkaloids, terpene, flavonoids, and flavanol glycosides	Anti-parasitic, antituberculosis anticancer, antiviral, antidiabetic, sexually transmitted infections, typhoid fever, antihypertensive	Anti-inflammatory, cardio-protective, neuro-protective, hepato-protective	Influenza A virus, new castle disease virus, herpes simplex virus, Epstein-Barr virus, hepatitis B virus, and foot-and-mouth disease virus in cloven-footed animals	[160,161,162,163,168,210]
*Zingiber officinale* R. (Fam Zingiberaceae) [Ginger]	Influenza, cough, sore throats, arthritis, lung diseases, peptic ulcer disease, hypertension, infectious diseases	Steroids, phenols, alkaloids, gingerols, zingerone, zingiberol, paradols, and shogaols	Antiarthritic, anticancer, antioxidant, antirhinoviral, antimicrobial, antiglycemic	Antioxidant, anti-inflammatory	Enterovirus 71, Japanese encephalitis virus, Epstein-Barr virus, herpes simplex virus types 1 and 2, influenza A virus, human immunodeficiency viruses, coronavirus SARS-CoV-1, rhinovirus, chikungunya virus, respiratory syncytial virus	[92,173,174,175,176,177,178,182,186]
*Momordica charantia* (Fam. Cucurbitaceae) [Bitter melon]	Diabetes, treat viral infections, toothache, diarrhea, gastrointestinal infections, ritual purposes	Minerals, vitamins, phenol compounds, triterpene, lipid, protein, glycosides, steroids, saponins, flavonoids	Antiviral, recurrent respiratory tract infections, anthelmintic, anticancer, antidiabetic, abortifacient, contraceptive	Antioxidant	HSV-1 and SINV viruses, HIV-1	[194,195,196, 198]
*Curcuma longa* L. (Fam. Zingiberaceae) [Tumeric]	Conjunctivitis, smallpox, sinusitis	Curcuminoids, Quercetin, Cuscumin, zingiberine, borneol, alpha phellandrene	Antioxidant, anticancer, immunomodulatory, antimicrobial, antiviral	Antioxidant, immune modulator	HIV-1, H1N1, hepatitis C, parainfluenza virus type-3, H6N1, human papillomavirus, and coxsackievirus B3	[205,206,207, 209]

## Data Availability

Not applicable.
